# Effect of Thermal Treatment on the Structure and Morphology of Vanadium Doped ZnO Nanostructures Obtained by Microwave Assisted Sol–Gel Method

**DOI:** 10.3390/gels8120811

**Published:** 2022-12-10

**Authors:** Cristina Maria Vlăduț, Oana-Cătălina Mocioiu, Silviu Preda, Jeanina Pandele-Cusu, Veronica Bratan, Roxana Trusca, Maria Zaharescu

**Affiliations:** 1 Institute of Physical Chemistry Ilie Murgulescu of the Romanian Academy, 202 Splaiul Independenţei, 060021 Bucharest, Romania; 2 Department of Science and Engineering of Oxide Materials and Nanomaterials, Faculty of Applied Chemistry and Materials Science, University Politehnica of Bucharest, 011061 Bucharest, Romania

**Keywords:** V doped ZnO, microwave sol–gel method, FTIR, thermal treatment, XRD, UV-VIS

## Abstract

In this paper, we conducted a fundamental study concerning the effect of thermal treatment on the structure and morphology of 2 mol% vanadium doped ZnO nanopowders obtained by microwave assisted sol–gel method (MW). The samples were analyzed by DTA, FTIR, XRD, SEM, and UV–Vis spectroscopy. The DTA results showed that above 500 °C, there was no mass loss in the TG curves, and ZnO crystallization occurred. The XRD patterns of the thermally treated powders at 500 °C and 650 °C showed the crystallization of ZnO (zincite) belonging to the wurtzite-type structure. It was found that in the 650 °C thermally treated powder, aside from ZnO, traces of Zn_3_(VO_4_)_2_ existed. FTIR spectra of the annealed samples confirmed the formation of the ZnO crystalline phase and V–O bands. The micrographs revealed that the temperature influenced the morphology. The increase in the annealing temperature led to the grain growth. The SEM images of the MW powder thermally treated at 650 °C showed two types of grains: hexagonal grains and cylindrical nanorods. UV–Vis spectra showed that the absorption band also increased with the increasing temperature of thermal treatment. The MW sample annealed at 650 °C had the highest absorption in ultraviolet domain.

## 1. Introduction

The design and fabrication of oxide nanostructures with a variety of shapes and sizes, correlating with novel features and prospective uses, has been the subject of intense research in recent decades [1]. For the synthesis of nanostructured materials beginning with solid, liquid, or gaseous phases, significant advancements have been realized [2]. However, the use of high temperature, high pressure, hazardous chemicals, and prolonged reaction times limits the majority of these techniques [3,4].

Even though physical methods have a high degree of reproducibility, chemical methods are more frequently used to make oxide nanostructures because of their advantages over physical ones including the ability to tailor the physical and chemical characteristics of the finished product based on the initial materials and the experimental conditions, low synthesis temperature, and homogeneous molecular mixing of the precursors [5,6].

Among the several chemical techniques, the sol–gel approach has grown in popularity in the field of materials science due to its adaptability in assuring the high purity and uniformity of the final products. Furthermore, it enables the insertion of large amounts of dopants, resulting in the controlled shape and size distribution of the generated nanomaterials [7,8,9].

Increased attention has recently been paid to the synthesis of functional nanomaterials using microwave energy [10,11]. When compared to traditional heating methods, the microwave (MW) assisted sol–gel technology is stated to be a faster, easier, cheaper, more energy-efficient, and effective process [12,13,14]. The employment of microwaves is suitable for the technological field since it decreases the reaction time from days or hours to minutes, improves the properties of synthesized nanostructures, and facilitates the formation of oxide nanocrystalline films on various substrates [3,15,16].

Microwaves provide fast and uniform heating because they may reach a depth dependent on the material’s dielectric characteristics, whereas conventional heating techniques are too sluggish to prevent inhomogeneity by transporting heat from the surface to the core material or solution [17].

As a result, the precursors instantly disintegrate, and a supersaturated solution is created. Therefore, important characteristics of the systems exposed to microwaves [18,19,20] as well as determinant synthesis conditions for creating well-defined materials (e.g., monodispersed nanoparticles needing quick and brief nucleation in a supersaturated solution) can be determined experimentally [18].

A narrow particle size distribution, enhanced purity, fewer surface defects, good shape, and morphological consistency, for instance, may all be provided by the employment of microwaves in the creation of nanoparticles while also speeding up the reaction rate [21]. More importantly, altering the reaction temperature, time, and system composition can have an impact on the crystallization [18,22].

Doping ZnO with transition metals results in new and enhanced properties, which have lately been explored and published by numerous researchers in diverse disciplines such as magnetic memories, gas sensors, diluted magnetic semiconductors, spintronics, and storage energy applications [23,24,25,26]. Vanadium (V) doping of ZnO has been found to give new features to the material including ferromagnetism, ferroelectricity, enhanced electrical and optical properties, and higher piezoelectric constants [24,27]. However, in the literature the effect of vanadium on the microstructural [23,24,28] and the optical properties of ZnO have led to some conflicting results [28,29,30,31,32,33,34,35]. 

Because of the numerous uses of V doped ZnO and the lack of publications on the preparation of V doped ZnO nanopowders, we provide here a study on the influence of thermal treatment and the synthesis method on the microstructural, morphological, and optical properties. The materials were obtained through an economical approach by the microwave (MW) assisted sol–gel method and the results were compared with the classical sol–gel method.

In a previous work, the authors studied 2 mol% V doped ZnO films and 2 mol% Mn doped ZnO films and revealed that the ones with vanadium had better piezoelectric properties [36]. Thus, in this paper, an in-depth study of the structure and morphology of the 2 mol% V doped ZnO powder as well as the influence of the synthesis method and the thermal treatment on the properties was approached.

## 2. Results and Discussion

### 2.1. As-Prepared Samples

Utilizing the microwave-assisted sol–gel technique (MW), white-pink solutions were produced. The precursors (see Section 4) were homogenized and subjected to microwaves for 10 min at 60 °C, 200 W power and 2.45 GHz frequency. The samples that resulted from letting the solutions gel at room temperature were compared to those prepared by the classical sol–gel procedure (SG).

#### 2.1.1. Differential Thermal Analysis

**Figure 1:**
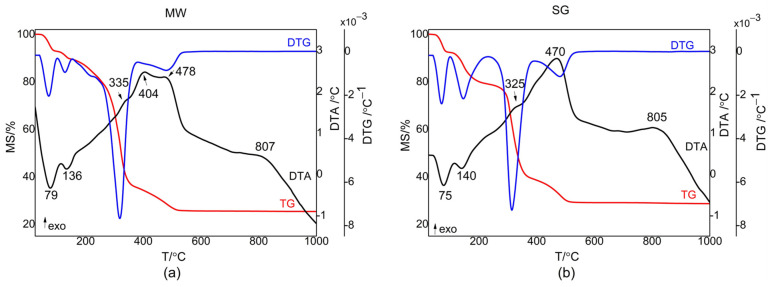
ATD, DTG, and TG curves for the V doped ZnO gels obtained by: (**a**) microwave assisted sol–gel method (MW), (**b**) sol–gel method (SG).

Differential thermal analysis and thermogravimetric analysis were used to investigate the thermal behavior of the synthesized gels. Figure 1 shows the results obtained for the MW and SG gels. 

The DTA curve in Figure 1a for the MW gel revealed five effects including two endothermic effects at 79 °C and 136 °C, and three exothermic effects at 335 °C, 404 °C, and 478 °C. The TG curve for the MW gel indicated a weight loss of around 74.1 percent up to 550 °C, while the DTA curve in Figure 1b for the SG gel showed four effects—two endothermic effects at 75 °C and 140 °C and two exothermic effects at 325 °C and 470 °C. The TG curve showed a weight loss of approximately 70.7 percent up to 550 °C. With a corresponding mass loss of around 10.55 percent for MW and 19.37 percent for SG, the removal of solvent groups and physically absorbed water occurred in the first two steps in both cases. Two minor endothermic effects, with minimums at about 70 °C and 140 °C accompanied these decompositions. The chemically bonded organics were removed from the gels in the third phase (around 190 °C and 390 °C for both samples), with a corresponding mass loss of about 53% for MW and 41% for SG. This exothermic reaction had an effect at around 330 °C. The structural hydroxyls and the remaining organics were removed in the fourth phase (between 390 and 550 °C), with a mass loss of 10.3% for MW and two exothermal effects at 404 °C and 478 °C, respectively. For the SG sample, the mass loss was 9.94%, having an exothermal effect at 470 °C. At higher temperatures above 550 °C, there was a thermal effect noticed on the DTA curves, an exothermal effect at around 800 °C, which was assigned to ZnO crystallization. Table 1 provides a summary of the breakdown ranges of the investigated gels, the mass losses, the accompanying thermal effects, and their assignments.

#### 2.1.2. Fourier Transform Infrared Spectroscopy (FTIR)

**Figure 2:**
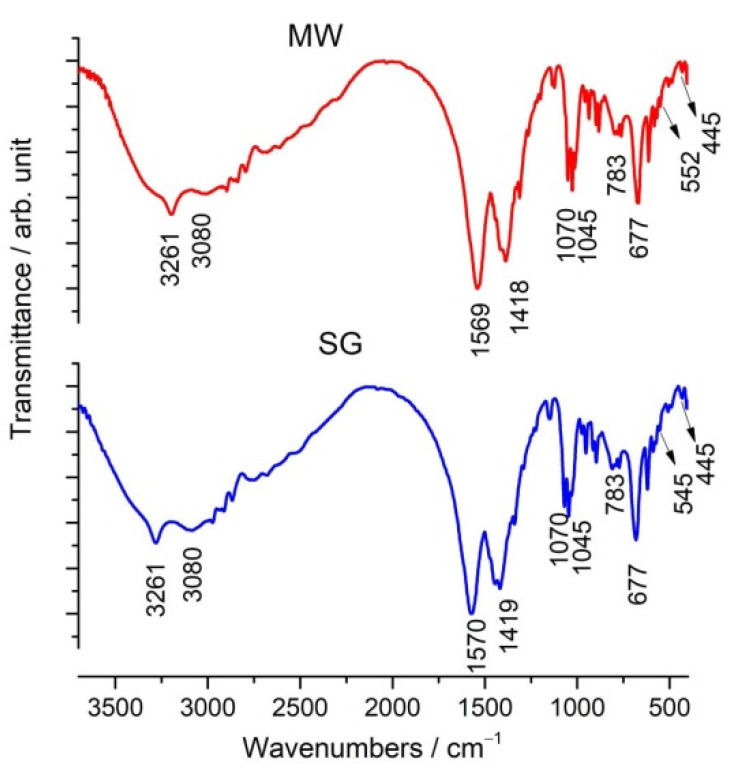
FTIR spectra of the V-doped ZnO gels obtained by the microwave assisted sol–gel method (MW) and the sol–gel method (SG).

Figure 2 shows the FTIR spectra of the V doped ZnO gels obtained by the microwave assisted sol–gel method (MW) and the classic sol–gel method (SG). The bands in the 3600–2000 cm^−1^ region corresponded to the vibration mode of the organic groups such as the CH_3_, CH_2_ as well as OH group from alcohols [37,38,39,40,41]; these overlapped into a wide band identified in the FTIR spectra of gels. Two bands at 1419 cm^−1^ and 1570 cm^−1^ were assigned to symmetric and asymmetric stretching of the C=O bond in the acetate group. The bands at 1045 cm^−1^ and 1070 cm^−1^ were characteristic to the symmetric and asymmetric stretching modes of the C–O bond in the acetate group. Below 677 cm^−1^, bands could be seen due to the Zn–O vibration [37,38,39,40,41]. In the spectra of the samples, the Zn–OH band was observed at 677 cm^−1^, and the bands at 502 and 445 cm^−1^ were attributed to Zn–O vibration. The small band at 1123 cm^−1^ was characteristic of V=O absorption, as reported by Ali [28]. The bands at 783 cm^−1^ and 545 (552) cm^−1^ were attributed to the stretching vibrations of the V–O–V bands, respectively [42,43]. The hydroxyl group at 3410 cm^−1^ and the bands associated with the V-doped ZnO at 780 cm^−1^ and 650 cm^−1^ in V:ZnO obtained by the sol–gel method were previously reported [28,44].

### 2.2. Thermally Treated Samples

Based on the results obtained by thermal analysis, the gels were thermally treated at 300 °C, 500 °C, and 650 °C for 1 h.

#### 2.2.1. X-ray Diffraction (XRD)

**Figure 3:**
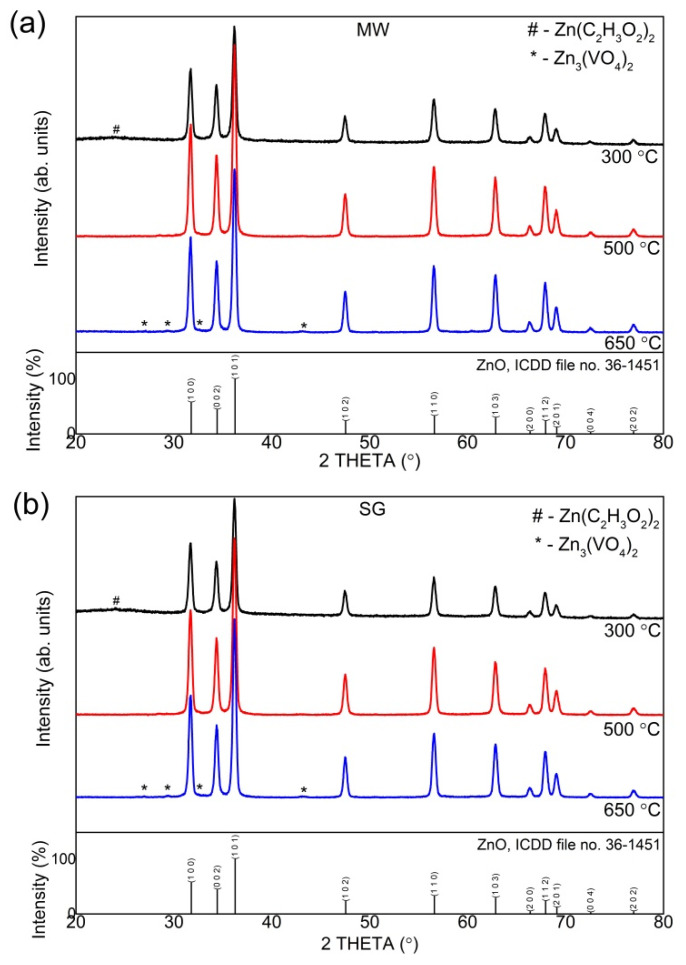
XRD patterns of the thermally treated samples prepared by: (**a**) microwave assisted sol–gel method (MW), (**b**) sol–gel method (SG).

The structure of the thermally treated powders (at 300 °C, 500 °C, and 650 °C for 1 h) was investigated by XRD. The XRD patterns are shown in Figure 3a,b. The crystallization behavior of the powders was similar, regardless of the preparation method. Both samples at 300 °C still had traces of the precursor (zinc acetate) and amorphous phase, in the presence of a well-crystallized phase. The crystalline phase matched well against ICDD file no. 36-1451, corresponding to ZnO, zincite. ZnO belongs to the wurtzite-type structure and crystallizes in the hexagonal P6_3_mc space group. The precursor completely transforms at 500 °C and no amorphous phase is detected. Both samples at 500 °C showed the zincite single phase, polycrystalline, and randomly oriented. At this temperature, no other phases (V-based compounds) were detected within the limit of the instrument, suggesting that the vanadium is incorporated into the zincite lattice or is well-dispersed in an amorphous state (not detected by XRD due to small amount) on the surface of the zincite crystallites. At 650 °C, traces of secondary phase(s) were detected in both powders. The thermal treatment usually leads to changes in the unit cell data and crystallite size, but this was not observed for the powders treated at 650 °C (Table 2), leading to a higher probability that the vanadium-based compound is in an amorphous state. The incorporation of a dopant into the zincite crystal structure should modify the lattice constants [29,45]. The presence of an amorphous phase containing vanadium-based compounds at the surface of the crystallites could lead to the formation of the new crystalline phase during thermal treatment [46]. According to other groups [30,31,32], the possible secondary phases could be V_2_O_3_, VO_2_, V_2_O_5_, Zn_3_(VO_4_)_2_, and Zn_2_V_2_O_7_. According to the phase equilibria diagrams in [47] and [48], the most probable phase composition of the powders thermally treated at 650 °C is a mixture of ZnO and Zn_3_(VO_4_)_2_.

Table 2 lists the lattice constants and the average crystallite size. The average crystallite size was determined by the Williamson–Hall method [49], and calculated from the integral line width. Plotting $\frac{\beta \cos\theta }{\lambda }$ against $\frac{\sin\theta }{\lambda }$ makes it possible to obtain crystallite size *L* from the gradient of the approximation line.

The lattice constants, listed in Table 2, were close to the indexed values (ICDD file no. 36-1451). Moreover, a slight decrease in the lattice constants can be noticed, particularly on the c-axis. The thermal treatment was accompanied by microdefect healing (vacancies, holes, interstitial impurities, dislocations), structure densification, and energetic tendency to the equilibrium state [50]. The improvement in the crystallinity could also be noticed from the full-width at half-maximum (FWHM) values, which were narrower as the thermal treatment temperature increased.

#### 2.2.2. Scanning Electron Microscopy (SEM)

**Figure 4:**
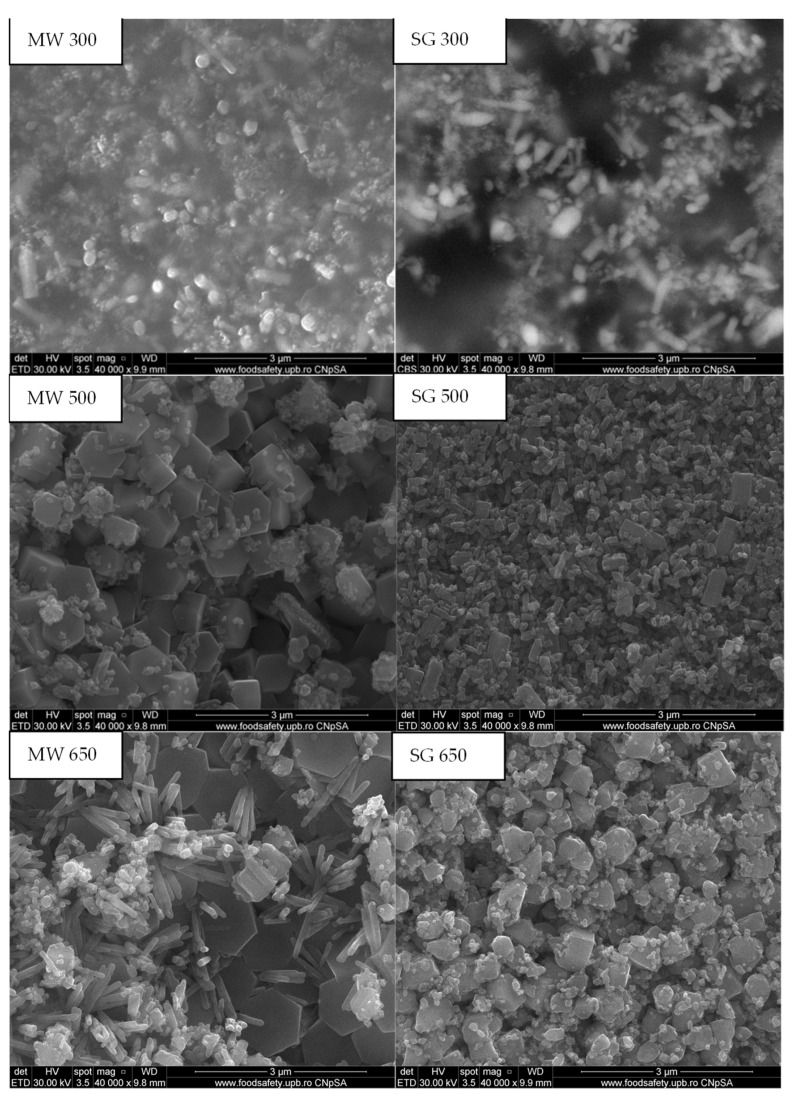
SEM images of the MW and SG powders, thermally treated at 350 °C/1 h, 500 °C/1 h, and 650 °C/1 h.

**Figure 5:**
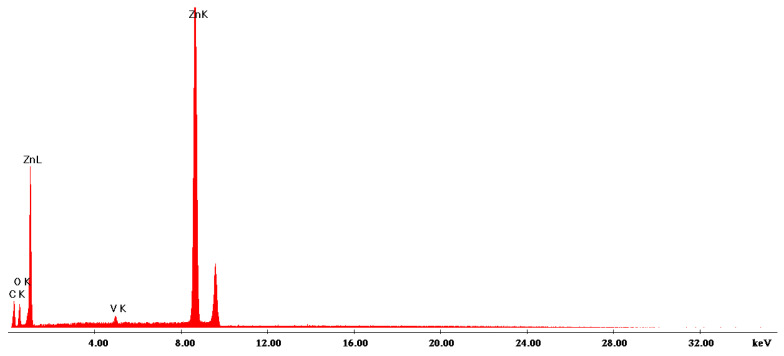
EDX spectra of the MW powders thermally treated at 650 °C/1 h.

The morphology of the thermally treated powders was investigated using the SEM technique and is illustrated in Figure 4. The micrographs revealed that not only does the temperature influence the morphology, but also the synthesis method. The SEM images of powders treated at 300 °C showed the formation of some nanocrystals and a hazy zone between the grains could be observed, which can be attributed to the amorphous material, thus being in agreement with the XRD patterns. The increase in the annealing temperature led to the grain growth. MW powders treated at 500 °C had a well-defined shape, consisting mostly of hexagonal grains, while the SG ones were comprised of a mixture of hexagonal, cubical, and spherical grains. This correlates with the increase in crystallinity in the XRD patterns. The particle size of these nanostructures was found to be within the range of 30–250 nm for MW and 30–170 nm for SG. The SEM images of the powders thermally treated at 650 °C showed grains with different dimensions and morphologies. In the case of the SG powder thermally treated at 650 °C, the grains had cuboidal shapes and dimensions between 43 and 316 nm. The SEM image of the MW powder thermally treated at 650 °C revealed that there were two types of grains: one hexagonal of 80–200 nm and the second cylindrical nanorods with the diameter of 29–76 nm. The hexagonal crystals could be attributed to the ZnO crystalline phase from the XRD pattern and the nanorod ones could be attributed to the Zn_3_(VO_4_)_2_ crystalline phase identified in the XRD. The energy dispersive X-ray spectroscopy (EDX) analysis (Figure 5) confirmed the presence of vanadium in the MW powder thermally treated at 650 °C.

#### 2.2.3. Fourier Transform Infrared Spectroscopy (FTIR)

**Figure 6:**
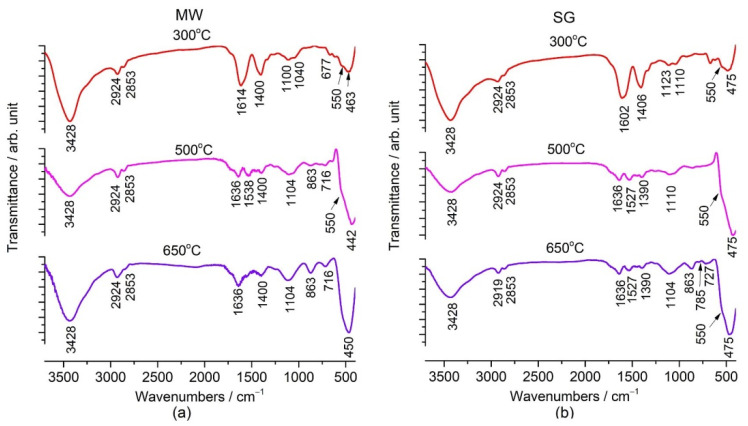
FTIR spectra of the thermally treated powders at 300 °C, 500 °C, and 650 °C for: (**a**) the microwave assisted sol–gel method (MW) and (**b**) the sol–gel method (SG).

In Figure 6, the FTIR spectra of the thermally treated powders at 300 °C, 500 °C, and 650 °C are presented. In Figure 6a are presented the FTIR spectra for powders obtained by the microwave assisted sol–gel method, while Figure 6b shows the spectra for the sol–gel obtained powders for comparison.

The spectra of our powders treated at 300 °C showed bands characteristic to organic species: at 3428 cm^−1^ hydroxyl groups; at 2924 cm^−1^ and 2853 cm^−1^ CH_2_ groups; at 1400 cm^−1^ and 1614 cm^−1^, two small bands for stretching of C=O bond in the acetate groups. The bands in the low frequency region at 463 cm^−1^ in MW and 475 cm^−1^ in SG corresponded to the lattice vibration mode of Zn–O, and the shoulder at 550 cm^−1^ was characteristic of the V–O–Zn vibration. In the literature, few data can be seen. Zargar et al. [33] reported the Raman spectrum of a V doped ZnO thick film thermally treated at 550 °C with a band at 435 cm^−1^ attributed to the ZnO 𝐸2 (high) phonon mode, and the band at 578 cm^−1^ was assigned to the ZnO 𝐸1(LO) mode. Relevant changes in the FTIR spectrum of the powders thermally treated at 650 °C could be seen by the appearance of a structured absorption band at 450 cm^−1^ assigned to the Zn_3_(VO_4_)_2_ phase. Vanadium infusion into the sub-lattice with a tetrahedral coordination of ZnO was clearly illustrated by the bands appearing at 727 cm^−1^, 785 cm^−1^, and 863 cm^−1^. Other researchers [33,34,51,52] have reported similar bands. 

#### 2.2.4. UV–Vis Spectroscopy

**Figure 7:**
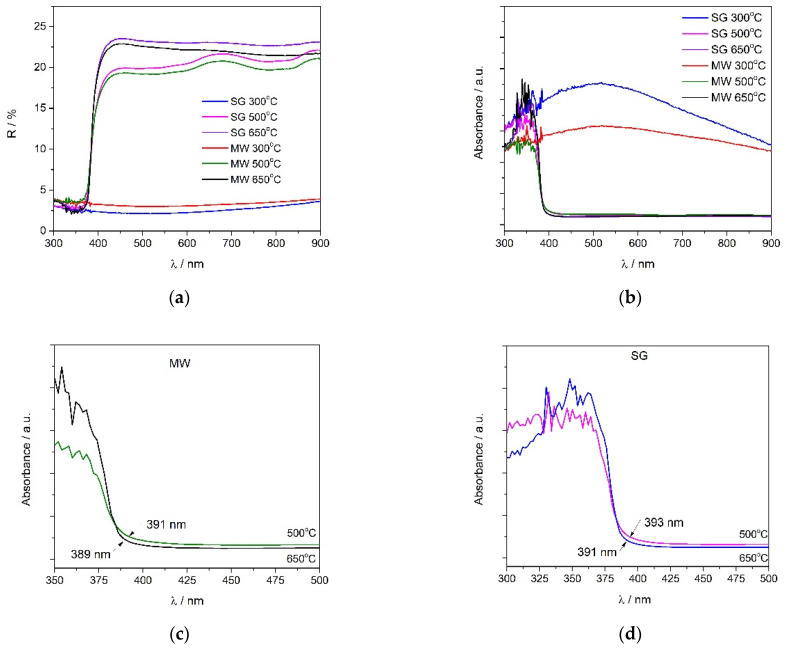
UV–Vis: (**a**) reflectance spectra; (**b**) absorbance spectra of the thermally treated powders at 300 °C, 500 °C, and 650 °C; (**c**) absorbance spectra in the 350–500 nm range for the microwave assisted sol–gel method (MW); and (**d**) absorbance spectra in the 350–500 nm range for the sol–gel method (SG).

UV–Vis spectroscopy was used to follow the formation of zinc oxide. The obtained spectra are shown in Figure 7. From Figure 7a,b it can be observed that the temperature of the thermal treatment had a significant influence. V doped ZnO thermally treated at 300 °C presented completely different spectra, with the reflectance values being low on the whole domain, and not only in the UV region as expected. This behavior could be due to the specular reflectance, which could be higher in this case because of the poor crystallization of zinc oxide. However, based on the XRD data, light absorption due to the presence of different vanadium compounds and/or to the presence of the organic precursor cannot be neglected either [53,54]. Increasing the thermal treatment temperature led to obtaining the characteristic spectra of ZnO. The powders presented a strong absorption at a wavelength lower than 400 nm due to the electronic transitions from the valence to the conduction band [35,55]. Figure 7c,d shows the spectra obtained for the 350–500 nm region, in order to highlight the effect of temperature and to determine the cutoff wavelength needed to calculate the band gap energies. For this purpose, the E = hc/λ_cut-off_ formula was used. The obtained values were 3.17 eV and 3.19 eV for the MW powders at 500 °C and 650 °C, respectively. The band gap for the SG powders at 500 °C was 3.15 eV and for the SG powders at 650 °C it was 3.17 eV. The difference between the pattern of the spectra and the band gap energy values could be related to the difference in the size and morphology of the particles [56,57]. The low reflectance of these samples in the visible region (Figure 7a) was probably due to the specular reflection and/or to the presence of vanadium, as reported below in the literature [29,58].

## 3. Conclusions

In this paper, the results regarding the effect of thermal treatment on the structure and morphology of 2 mol% vanadium doped ZnO nanostructures obtained by the microwave assisted sol–gel method (MW) are presented. The obtained results were compared with the samples obtained by the classical sol–gel method (SG). The TG/DTG/DTA results show that the thermal decomposition of both the MW and SG samples was finished at around 500 °C. The FTIR spectra of the gels showed the formation of hydroxyzincite and the presence of the V–O–V and V=O bands. In the case of thermally treated powders at 300 °C, the XRD patterns showed ZnO crystallization and the presence of an amorphous phase also confirmed by the FTIR and SEM results. For the powders thermally treated at 500 °C and 650 °C, the XRD patterns showed the crystallization of ZnO (zincite) belonging to the wurtzite-type structure. The powder thermally treated at 650 °C presented ZnO and traces of Zn_3_(VO_4_)_2_. The FTIR spectra of the thermally treated samples showed the formation of the crystalline phase of ZnO by the structure of the bands at 450/475 cm^−1^. Thus, the bands of V–O were identified as a shoulder at 550 cm^−1^ and as small bands in the 600–1000 cm^−1^ range. 

The micrographs revealed that not only does the temperature influence the morphology, but also the synthesis method. At 300 °C, we could see the formation of some nanocrystals as well as a hazy zone between the grains, which could be attributed to the amorphous material. The increase in the annealing temperature led to the grain growth. Powders treated at 500 °C obtained by the MW assisted sol–gel method had a well-defined shape, consisting mostly of hexagonal grains, while the powders obtained by the sol–gel method formed a mixture of hexagonal, cubical, and spherical grains. In the SG powder thermally treated at 650 °C, the grains had cuboidal shapes while the MW sample showed two types of grains: hexagonal grains and cylindrical nanorods. 

UV–Vis revealed that the absorption band increased with the increasing temperature of the thermal treatment, which was related to a higher crystallinity of the resulted samples. Thermally treated MW powders at 650 °C had the highest absorption in the ultraviolet domain. 

## 4. Materials and Methods

### 4.1. Synthesis

Precursor solutions were obtained from the following starting ingredients, all of which were of reagent grade: Zinc acetate dihydrate—Zn(CH_3_COO)_2_·2H_2_O (Merck), Vanadyl acetylacetonate—OV(C_5_H_7_O_2_)_2_, absolute ethanol—C_2_H_5_OH, and triethanolamine (TEA)—C_6_H_15_NO_3_. 

A 2 mol% vanadium doped ZnO solution was prepared by combining the precursors in absolute ethanol at 50 °C for 15 min, then gradually adding triethanolamine drop by drop in a molar ratio of ZnAc:TEA = 5:1. The final product was subjected to microwaves for 10 min at 60 °C, 200 W power, and 2.45 GHz frequency. In the case of the classical sol–gel method (SG), the precursors were homogenized at 50 °C for two hours on a heated plate, without being exposed to microwaves.

Gelation took place at ambient temperature. All of the resulting gels were dried at 100 °C for 24 h before being thermally treated for one hour at three different temperatures: 300 °C, 500 °C, and 650 °C.

### 4.2. Methods of Characterization

A Nicolet 6700 instrument was used to record the Fourier Transform Infrared Spectra (FTIR) for the gels and thermally treated powders in the 400–4000 cm^−1^ domain. The KBr pellets were used to record the spectra in transmittance mode. The measurements had a 4 cm^−1^ sensitivity. The qualitative interpretation based on the normalized spectra was carried out.

Using Mettler Toledo TGA/DTA 851e equipment in a flowing air atmosphere of 80 mL/min and in Al_2_O_3_ crucibles, the thermal behavior of the synthesized gels was studied using differential thermal analysis (DTA) and thermogravimetric analysis (TGA). The heating rate was 10 degrees/min, and the maximum temperature was set at 1000 °C.

Scanning electron microscopy (SEM). The surface morphologies and compositional analysis of the calcined samples were evaluated by scanning electron microscopy (SEM-EDX) using a FEI Quanta 3D FEG instrument equipped with the Octane Elect EDS system. The powder samples were placed on double-sided carbon tape and recorded without aa coating at an accelerating voltage of 10 and 20 kV in high vacuum mode. 

X-ray diffraction (XRD) data were collected on a Ultima IV diffractometer (Rigaku Corp., Tokyo, Japan) using CuKα radiation (λ = 1.5406 Å), operated at 40 kV and 30 mA, over the 2θ range 20−80°, at a scanning rate of 2°/min, with a step width of 0.02°. The phase identification was performed by means of search/match in PDXL (Rigaku’s proprietary software), connected to the International Center for Diffraction Data (ICDD) PDF-2 database.

A Perkin Elmer Lambda 35 spectrophotometer with an integrating sphere was used to record the UV–Vis spectra. The powder was introduced in a special holder and the analysis was made in the wavelength domain of 900–300 nm, with Spectralon as the reference. Based on the Kubelka–Munk function, the collected reflectance data were transformed into absorption spectra. 

## Figures and Tables

**Table 1:** The TGA/DTA results for the V doped ZnO gels.

Sample	Temperature Range, (°C)	Thermal Effects, (°C)		Mass Loss, (%)	Assignments
		Endo	Exo		
MW	25–100	79		7.1	Physically absorbed water and solvent groups elimination
	100–190	136		3.45	Decomposition and elimination of organic species
	190–390		335	53	Burning out organic residues
	390–550		404 478	10.3	Elimination of the structural hydroxyl groups and transformation from amorphous to crystalline powder
	550–1000		807	0.3	Crystallization
				∑ = 74.1	
SG	25–100	75	-	7.65	Physically absorbed water and solvent groups elimination
	100–190	140	-	11.72	Decomposition, elimination of organic species and structural hydroxyl groups
	190–390	-	325	41	Elimination of the structural hydroxyl groups and burning out organic residues
	390–550	-	470	9.94	Elimination of the structural hydroxyl groups and amorphous powder crystallization
	550–1000	-	805	0.4	Crystallization
				∑ = 70.7	

**Table 2:** The lattice parameters and the average crystallite size.

Sample	Phase(s)	Lattice Constants				Crystallite Size L (nm)
		*a* (Å)	*c* (Å)	*α* (°)	*γ* (°)	
MW@300 °C	ZnO	3.2505(5)	5.2090(8)	90	120	19
	Zn(C_2_H_3_O_2_)_2_	traces				-
MW@500 °C	ZnO	3.2504(3)	5.2061(6)	90	120	20
MW@650 °C	ZnO	3.2505(3)	5.2055(6)	90	120	20
	Zn_3_(VO_4_)_2_	traces				
SG@300 °C	ZnO	3.2507(4)	5.2087(6)	90	120	18
	Zn(C_2_H_3_O_2_)_2_	traces				-
SG@500 °C	ZnO	3.2504(3)	5.2063(5)	90	120	20
SG@650 °C	ZnO	3.2502(3)	5.2050(5)	90	120	20
	Zn_3_(VO_4_)_2_	traces				-
ZnO (ICDD file 26-1451)		3.2500	5.207	90	120	-

## Data Availability

Not applicable.
